# Metabolomics Reveals Metabolically Healthy and Unhealthy Obese Individuals Differ in their Response to a Caloric Challenge

**DOI:** 10.1371/journal.pone.0134613

**Published:** 2015-08-14

**Authors:** Flavia Badoud, Karen P. Lam, Maude Perreault, Michael A. Zulyniak, Philip Britz-McKibbin, David M. Mutch

**Affiliations:** 1 Department of Human Health and Nutritional Sciences, University of Guelph, Guelph, ON, Canada; 2 Department of Chemistry and Chemical Biology, McMaster University, Hamilton, ON, Canada; University College Dublin, IRELAND

## Abstract

**Objective:**

To determine if metabolically healthy obese (MHO) individuals have a different metabolic response to a standardized diet compared to lean healthy (LH) and metabolically unhealthy obese (MUO) individuals.

**Methods:**

Thirty adults (35–70 yrs) were classified as LH, MHO, and MUO according to anthropometric and clinical measurements. Participants consumed a standardized high calorie meal (~1330 kcal). Blood glucose and insulin were measured at fasting, and 15, 30, 60, 90 and 120 min postprandially. Additional blood samples were collected for the targeted analysis of amino acids (AAs) and derivatives, and fatty acids (FAs).

**Results:**

The postprandial response (i.e., area under the curve, AUC) for serum glucose and insulin were similar between MHO and LH individuals, and significantly lower than MUO individuals (p < 0.05). Minor differences were found in postprandial responses for AAs between MHO and MUO individuals, while three polyunsaturated FAs (linoleic acid, γ-linolenic acid, arachidonic acid) showed smaller changes in serum after the meal in MHO individuals compared to MUO. Fasting levels for various AAs (notably branched-chain AA) and FAs (e.g., saturated myristic and palmitic acids) were found to correlate with glucose and insulin AUC.

**Conclusion:**

MHO individuals show preserved insulin sensitivity and a greater ability to adapt to a caloric challenge compared to MUO individuals.

## Introduction

The widespread availability of foods rich in refined carbohydrates and fats is a major contributor to the obesity epidemic [1]. As most of an individual’s day is spent in a postprandial state [2], studying a person’s response to a diet provides valuable insight into metabolic function. Further, the dynamic adaptation to a caloric challenge is highly informative. For example, postprandial increases in triglycerides (TG) are considered a risk factor for cardiovascular diseases (CVD) [3, 4], while high postprandial glucose and insulin levels indicate a risk for type 2 diabetes (T2D) [5]. Therefore, evaluating metabolic responses following a standardized meal challenge can provide a better understanding of the link between foods and metabolism compared to fasted markers, and has the potential to reveal differences in metabolic adaptability between individuals [6].

Recently, a subgroup of obese individuals, commonly referred to as metabolically healthy obese (MHO), was identified as being protected from the usual downstream complications associated with obesity, such as T2D and CVD. Studying this particular phenotype has revealed that MHO exhibit distinct molecular and metabolic characteristics compared to metabolically unhealthy obese (MUO) individuals [7, 8]. Previous reports have shown that the response to an oral glucose tolerance test (OGTT) [9–12] or an oral fat load differs between individuals varying in cardiometabolic risk [2, 4]. For example, a recent study reported that MHO co-twins had lower area under the curves (AUC) for insulin and glucose following an OGTT compared to MUO co-twins [12]. Perez-Martinez *et al*. showed that after an oral fat load, postprandial TG metabolism (i.e., the AUC for TG) and inflammatory status were lower in MHO individuals compared to their MUO counterparts, thus showing the greater ability of MHO to adapt to a caloric challenge [4]. Therefore, underlying differences in response to a dietary challenge is of high interest in order to better understand mechanisms linked to cardiometabolic health.

This study is the first to examine the glycemic and insulinemic responses to a standardized high-calorie meal in lean healthy (LH), MHO, and MUO individuals. We previously reported that circulating amino acid (AA) and fatty acid (FA) profiles differed between MHO and MUO individuals in a fasted state [13, 14]. Therefore, the present study also used a targeted metabolomics approach to evaluate if the metabolic adaptation to this challenge had a different effect on AA and FA profiles in MHO and MUO individuals. Finally, we hypothesized that studying AAs and FAs could lead to the discovery of metabolites that may potentially serve as predictors for a person’s postprandial glucose and/or insulin response to a caloric challenge. Together this report highlights the greater ability of MHO individuals to adapt to a high calorie meal compared to their MUO counterparts as well as their preserved insulin sensitivity.

## Materials and Methods

### Subjects and Study design

Serum and plasma samples were obtained from individuals participating in the Diabetes Risk Assessment study (Clinicaltrials.gov ID #NCT01884714). Persons were screened over the phone and excluded if they met any one of the following criteria: 1) aged < 35 or > 70 years; 2) diagnosed with an acute or chronic autoimmune inflammatory disease, infectious disease, viral infection, and/or cancer; or 3) regular alcohol consumption exceeding 2 drinks/day (1 drink = 10 g alcohol). None of the participants were taking antidiabetic medications. All participants signed a consent form and the research protocol was approved by the University of Guelph Research Ethics Board (REB#10AP033).

### Subject Classification

Thirty participants were classified into 3 distinct groups: LH (n = 10), MHO (n = 10), and MUO (n = 10) based on their adiposity and metabolic status, as previously described [14]. Briefly, adiposity status was determined using the revised BMI cut-offs proposed by Shah and Braverman, where lean was considered < 28kg/m^2^ for males and < 24kg/m^2^ for females, and obese was considered ≥ 28kg/m^2^ for males and ≥ 24kg/m^2^ for females [15]. Metabolic status was determined using criteria adapted from that originally proposed by Karelis *et al*. in order to account for sex-specific differences and medication [16]. An individual was considered “metabolically healthy” if 3 or more of the following criteria were met: HDL-cholesterol > 1.0mmol/L for males and > 1.3mmol/L for females; triglycerides < 1.7mmol/L without use of lipid-lowering drugs; total cholesterol < 5.2mmol/L; LDL-cholesterol < 2.6mmol/L; and HOMA-IR < 1.95 without use of anti-diabetic drugs. Each group was comprised of 7 women and 3 men. Complete details regarding the methodologies used to measure biochemical parameters and percentage body fat can be found in [14]. LH, MHO, and MUO groups were matched for age, while the MHO and MUO groups were matched for BMI and percentage body fat (S1 Table).

### Caloric Challenge

A high calorie meal (fast-food breakfast representative of the Western diet) was given to each participant following a 12 h overnight fast. The meal consisted of 2 sausage egg English muffins, 1 apple turnover, and ~370 mL of concentrated orange juice, and provided a total calorie intake of ~1330 kcal (i.e., 66 g of fat, 141 g of carbohydrates, 5 g of fibre and 42 g of proteins). The standardized meal was eaten within 20 min. Blood was collected for glucose and insulin measurements at fasting and postprandially at 15, 30, 60, 90 and 120 min. Additional blood samples were collected at fasting and at the 120 min postprandial time point (T120) for targeted analyses of AAs and derivatives, as well as FAs.

To limit potential confounding lifestyle factors, participants were asked to avoid rigorous exercise, over-the-counter medication, dietary supplements, vitamins, and herbal supplements for 48 h prior to the study visit. Furthermore, participants fasted for at least 12 h after consuming a standardized single-serving frozen dinner meal the night preceding the study visit, and 1 dinner roll (whole wheat or white), 1 vegetable side of their choice (corn, peas, broccoli, carrots, squash, zucchini, or green beans), 1 fruit (apple, orange, banana, peach, grapes, or melon), and 500 mL bottled water.

### Blood metabolite profiling

#### Amino acid profiling

Plasma samples (50 μL) were analysed using capillary electrophoresis coupled to mass spectrometry (CE-MS), as previously described [13]. Peak areas and migration times were normalized relative to the internal standard (IS) 3-chloro-L-tyrosine, and data were reported as absolute level (μM) and percent postprandial change (%PP) following the standardized meal intake, where %PP = ((PP value − fasting value) / fasting value) * 100).

#### Fatty acid profiling

FAs were profiled in serum samples using gas chromatography (GC), as previously described [14]. Briefly, 10 μl of a 1 μg/μL C17:0 internal standard was added to 100 μL of serum. FAs were extracted with chloroform:methanol (2:1, v/v) and methylated at 100°C for 1.5 hrs. All samples were analyzed on an Agilent DB-FFAP column (15m x 0.1 ID mm; 0.1 μm), using an Agilent Technologies 7890A GC system (Agilent Technologies, Mississauga, ON, Canada) with flame ionization detector. Peaks were identified by comparison to a panel of 49 FA methyl ester standards suspended in hexane (ranging from C8:0 to C24:1n-9). Relative FA values were calculated as a % of total peak area and data were reported as %PP (as described above) using relative FA values.

### Standardized Meal Composition Analysis

#### Protein hydrolysis for amino acid composition

Acid hydrolysis was performed on the homogenized meal to determine the AA composition. Briefly, the meal was homogenized in a standard blender and a 50 mg sample was placed in a glass tube to which 350 μL of 6 M hydrochloric acid (HCl) was added (n = 3). Samples were maintained at 100°C for 24 h under nitrogen, prior to being cooled to room temperature. The acid hydrolysate containing the meal sample was evaporated to dryness, then neutralized and reconstituted in 2.1 mL of 1M ammonium hydroxide (NH_4_OH). Ultrafiltration was performed using 3 kDa MWCO NanoSep centrifugal devices (Pall Life Sciences, Washington, NY, USA) at 13,000 g for 20 min. Prior to CE-MS analysis, 15 μL of the filtered hydrolysate was diluted to 50 μL in NH_4_Ac buffer (pH of 5) with a final concentration of 200 mM NH_4_Ac containing 25 μM of IS. AAs in the meal were expressed as relative abundances. Notably, during this procedure certain acid-labile amino acids have poor recoveries after protein digestion, including tryptophan and cysteine/cystine, while glutamine and asparagine are hydrolysed into glutamic acid and aspartic acid, respectively.

#### Fatty acid composition

An aliquot of 10 mg of the homogenized meal was used to determine FA composition by GC. 2.5 mL of 0.1 M KCl was added to 10 mg of the homogenized meal. The homogenate was transferred into 10 mL chloroform:methanol (2:1, v/v), and analyzed by GC using the same procedure as for the serum FA analysis. FAs were expressed as a relative abundance in the meal.

### Statistical Analyses

Statistical analyses were performed using GraphPad Prism 6 software (La Jolla, CA, USA). Non-parametric ANOVA Kruskal-Wallis tests were used to determine if the metabolites were statistically different between the three groups (p < 0.05). When significance was observed, a post-hoc non-parametric Mann-Whitney test was used for pairwise analyses (p < 0.05). AUC_total_ was calculated using the trapezoid rule, with a baseline of 0 over the range of fasting to T120 post meal intake. Separate regressions were fitted for all measured metabolites and insulin sensitivity indices using JMP 11 (SAS, Institute, Cary, NC), adjusted for sex, age and BMI. Significance was assessed after Bonferroni correction for multiple testing (p ≤ 0.01).

## Results

### Glycemic and insulinemic responses

**Fig 1:**
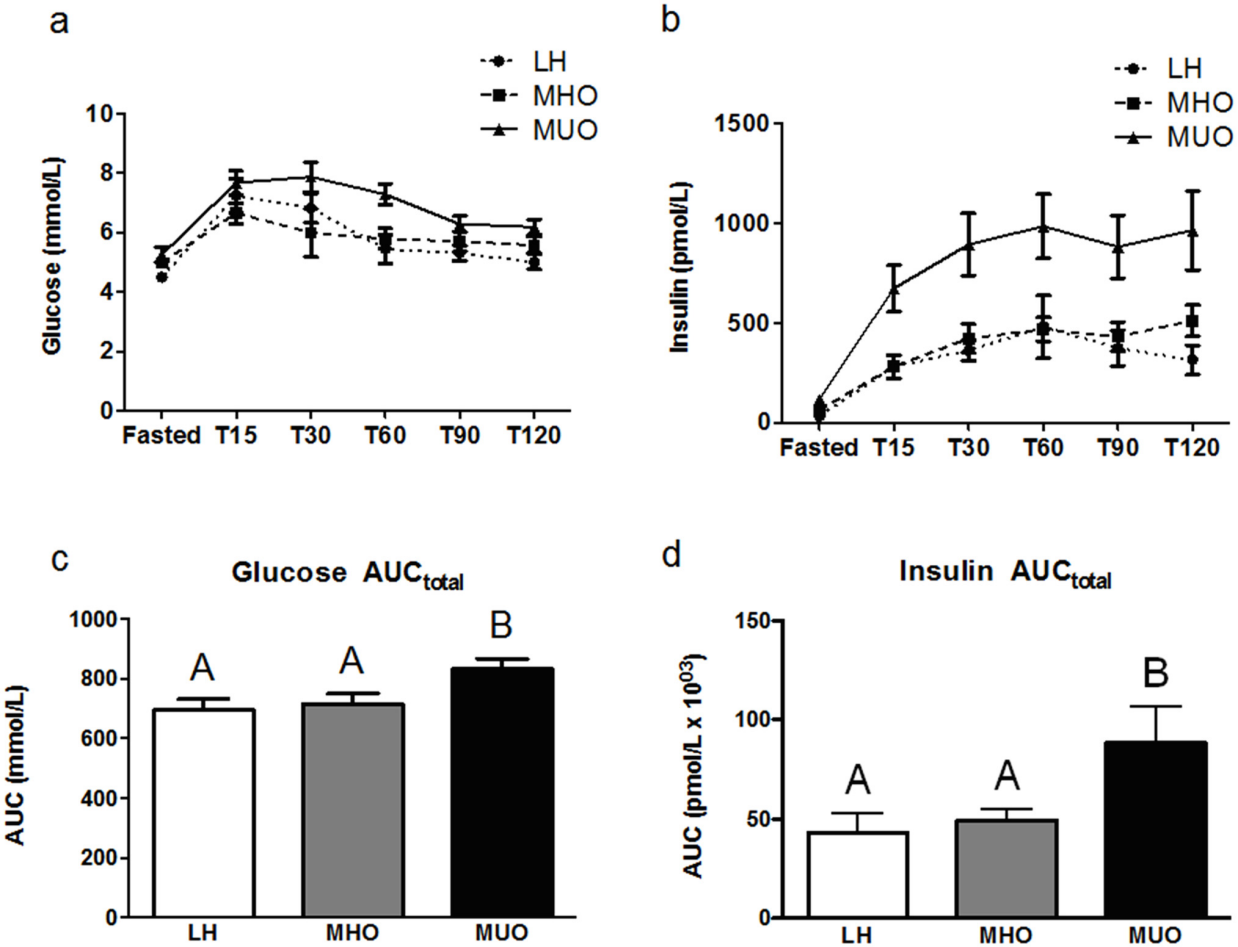
Postprandial serum glucose (a) and insulin (b) responses (mean ± SEM) following consumption of the standardized meal in lean healthy (LH; circle, n = 10), metabolically healthy obese (MHO; square, n = 10), and metabolically unhealthy obese (MUO; triangle, n = 10) individuals. Glucose (c) and insulin (d) Area Under the Curve (AUC_total_), where white bars = LH; grey bars = MHO; and black bars = MUO. A non-parametric ANOVA Kruskal-Wallis test followed by a post-hoc Mann-Whitney test was used to determine differences between groups. Bars not sharing the same letter are statistically different (p < 0.05).

Glucose and insulin AUC_total_ differed significantly between the three groups (Fig 1a and 1b). The AUC_total_ for glucose and insulin (Fig 1c and 1d) were not different between LH and MHO individuals (p = 0.40 and p = 0.31, respectively), but both values were significantly higher in MUO individuals (p = 0.02 between LH and MUO and p = 0.01 between MHO and MUO for glucose; and p < 0.01 between LH and MUO and p = 0.01 between MHO and MUO for insulin).

### Plasma amino acid and derivatives response

The levels of AAs and their derivatives were profiled in fasting and T120 plasma samples in the three distinct groups. A dataset comprising individual levels and ratios for the 39 AAs and their derivatives measured in each group are provided in S2 Table.

**Fig 2:**
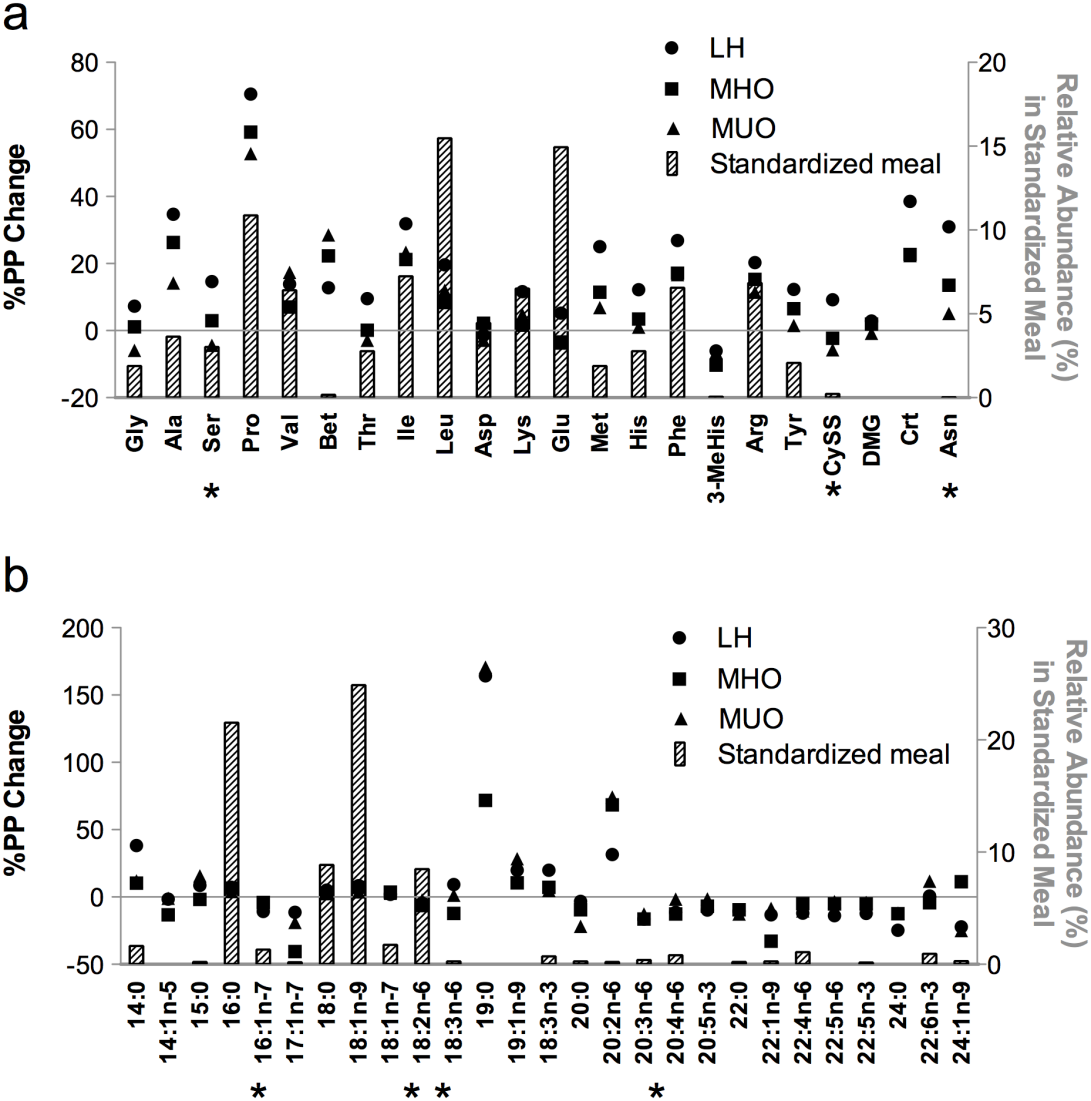
Amino acid (a) and fatty acid (b) composition of the standardized meal (grey bars, plotted on right y-axis). The corresponding %PP is plotted on the left y-axis for lean healthy (LH; circle, n = 10), metabolically healthy obese (MHO; square, n = 10) and metabolically unhealthy obese (MUO; triangle, n = 10) in plasma samples. Significant %PP (indicated by *) between the three groups were identified using a non-parametric ANOVA Kruskal-Wallis (p < 0.05) test followed by a post-hoc Mann-Whitney test (p < 0.05). Gly = glycine, Ala = alanine, Ser = serine, Pro = proline, Val = valine, Bet = betaine, Thr = threonine, Ile = isoleucine, Leu = leucine, Asp = aspartic acid, Lys = lysine, Glu = glutamic acid, Met = methionine, His = histidine, Phe = phenylalanine, MeHis = methylhistidine, Arg = arginine, Tyr = tyrosine, CySS = cystine, DMG = dimethylglycine, Crt = creatine, Asn = asparagine.

**Fig 3:**
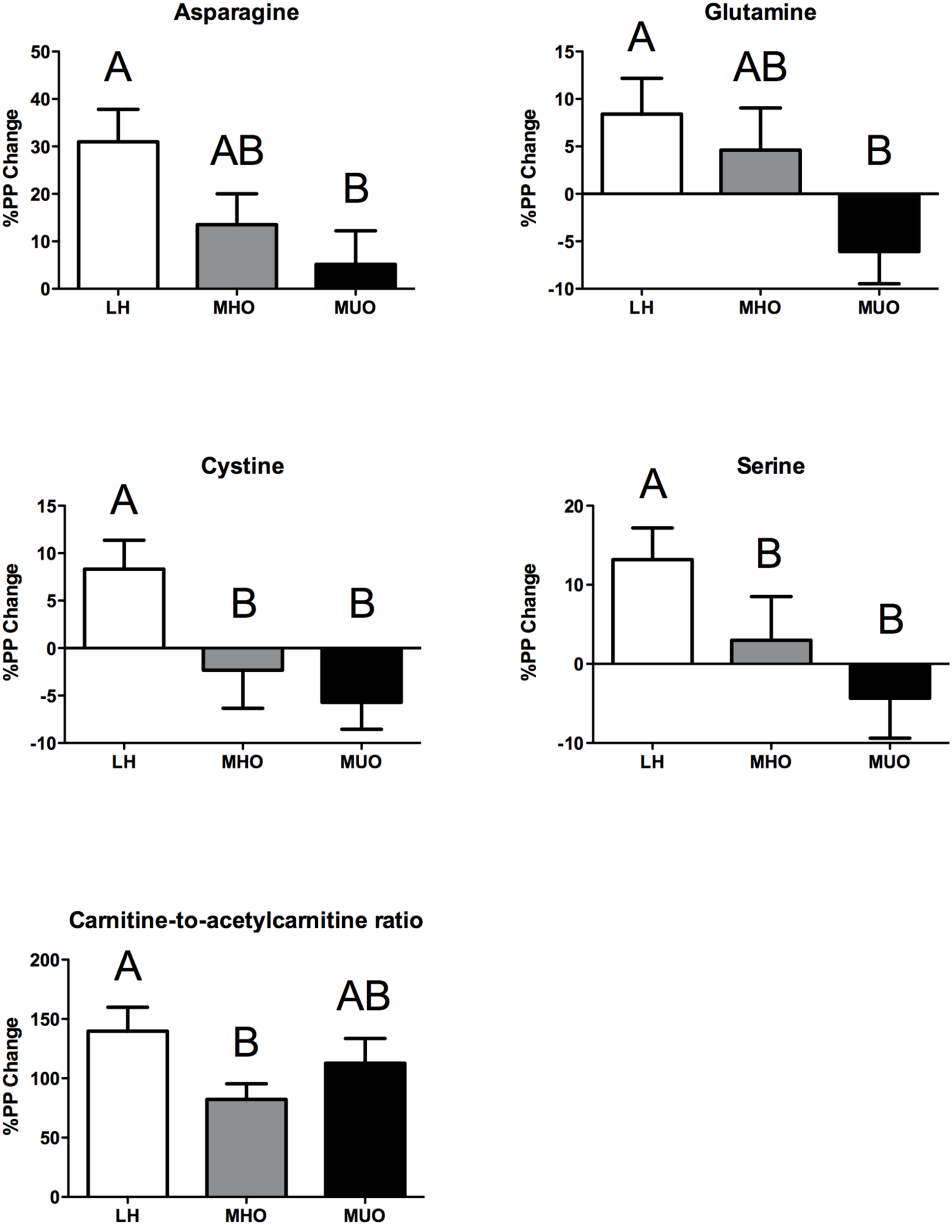
Mean %PP of plasma amino acid and derivatives measured by CE-MS for lean healthy (LH, n = 10, white bars), metabolically healthy obese (MHO, n = 10, grey bars) and metabolically unhealthy obese (MUO, n = 10, black bars). Significant %PP amino acids between the three groups were identified using a non-parametric ANOVA Kruskal-Wallis (p < 0.05) test followed by a post-hoc Mann-Whitney test (p < 0.05). Data is presented as mean %PP ± SEM.

We first determined the relative abundance of the detected AAs in the meal, and then overlaid this with the corresponding %PP change in plasma AAs for LH, MHO and MUO groups (Fig 2a). Branched-chain AAs (BCAA: leucine, isoleucine, and valine), glutamic acid + glutamine (i.e., glutamine is converted to glutamic acid during the acid hydrolysis procedure), and proline were the most abundant AAs found in the meal. Most of the AAs and derivatives were similarly increased within the 3 groups at T120 following the meal (e.g., alanine, proline), but several showed a reduction (e.g., acetylcarnitine, citrulline) (S2 Table). When examining the %PP change after the consumption of the standardized meal (Figs 2a and 3), four metabolites (i.e., asparagine, cystine, glutamine, and serine) and the carnitine-to-acetylcarnitine ratio were significantly different between the 3 groups (ANOVA Kruskal-Wallis p < 0.05, S2 Table). For asparagine and glutamine, the %PP change between fasting and T120 was intermediate for MHO individuals compared to MUO and LH, with levels in MUO being significantly lower compared to that of LH individuals. Cystine and serine revealed a significantly higher %PP change in the LH group relative to the obese groups, with no distinction between MHO and MUO. Finally, the carnitine-to-acetylcarnitine ratio was significantly increased for each group, where the %PP change was higher in LH compared to that of MHO individuals, while being intermediate for MUO individuals.

### Serum fatty acid response

**Fig 4:**
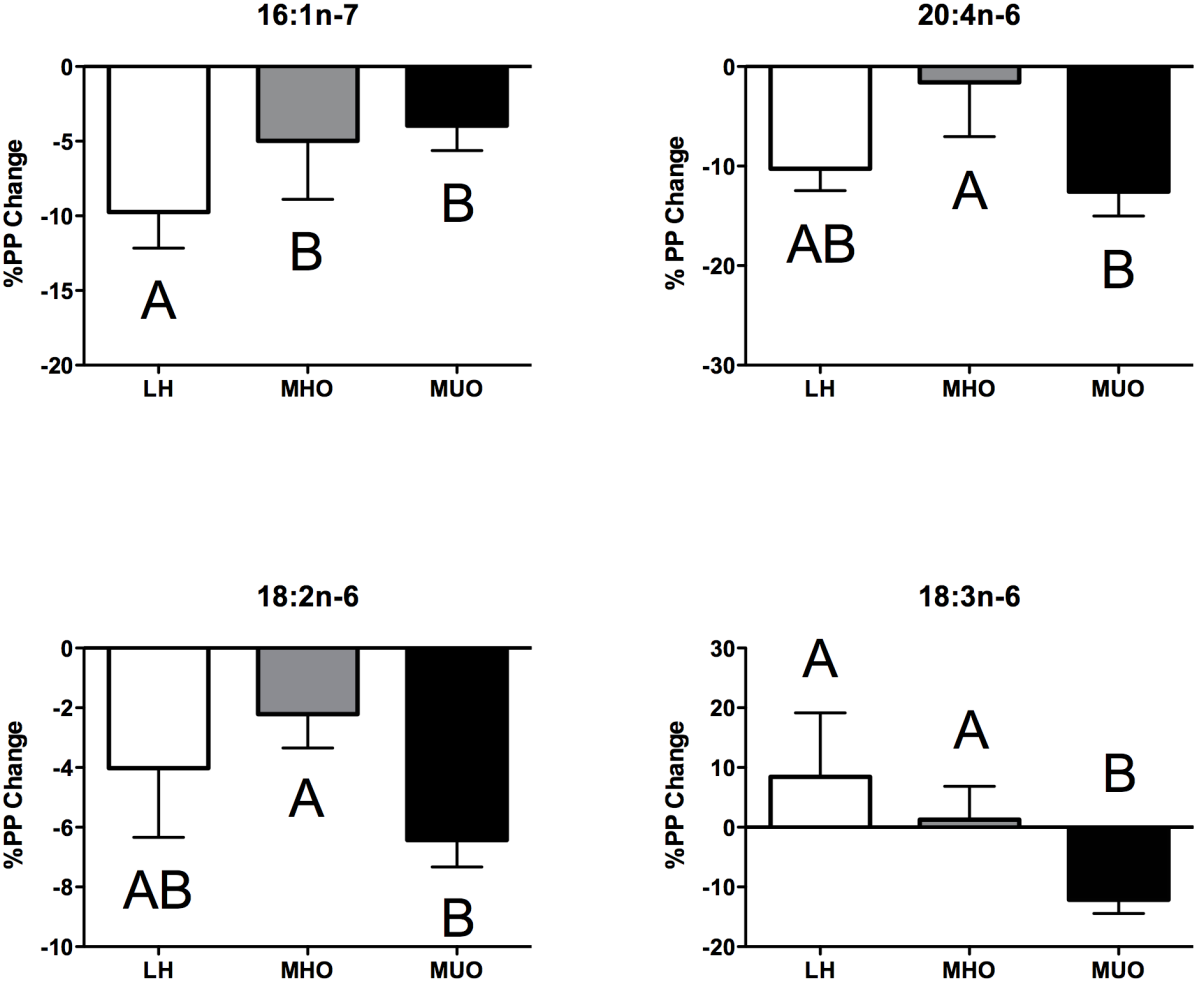
Mean %PP of serum fatty acids for lean healthy (LH, n = 10, white bars), metabolically healthy obese (MHO, n = 10, grey bars) and metabolically unhealthy obese (MUO, n = 10, black bars). Significant %PP between the three groups were identified using a non-parametric ANOVA Kruskal-Wallis test (p < 0.05) followed by a post-hoc Mann-Whitney test (p < 0.05). Data is presented as mean %PP ± SEM.

The standardized meal provided the most abundant dietary FAs, as expected (Fig 2b). Indeed, the meal contained ~38% saturated fat (SFA), of which palmitic acid (16:0) and stearic acid (18:0) were the most abundant, ~29% monounsaturated fat (MUFA) of which oleic acid (18:1n-9) was the most abundant, and ~17% polyunsaturated fat (PUFA), which consisted predominantly of linoleic acid (18:2n-6). In parallel, we profiled serum FAs in fasting and postprandial T120 plasma samples. Twenty-seven FAs were consistently detected in all samples and either increased or decreased similarly following the caloric challenge in the LH, MHO, and MUO groups (S3 Table). We also calculated the %PP change between the two time points. As shown in Fig 2b, which illustrates the abundance of FAs in the meal relative to the %PP between fasting and T120, four FAs (palmitoleic acid (16:1n-7), 18:2n-6, γ-linolenic acid (18:3n-6), and arachidonic acid (20:4n-6)) revealed significantly distinct %PP changes (Fig 4) between the three groups (ANOVA Kruskal-Wallis p < 0.05, see S3 Table). The %PP for 16:1n-7 was significantly lower in MHO and MUO compared to LH, while the %PP for 18:2n-6 and 20:4n-6 was significantly lower in MHO compared to MUO, but was intermediate for LH. In contrast, the %PP for 18:3n-6 was higher for LH and MHO in comparison with the MUO group.

### Baseline metabolites associate with measures of insulin sensitivity

We next examined the associations between all measured metabolites with markers of insulin sensitivity at both fasting (i.e., HOMA-IR, fasting glucose and insulin) and postprandially (i.e., glucose and insulin AUCs) to evaluate if metabolites correlated with indices of insulin sensitivity and if they could be used to potentially predict glucose and insulin responses (Table 1). None of the AAs that showed a significantly different %PP change between the 3 groups correlated with markers of insulin sensitivity. However, we observed positive associations between other AAs and their derivatives with insulin sensitivity indices. Proline and leucine were positively associated with HOMA-IR; creatine and proline with fasting glucose; and proline and leucine with fasting insulin. Moreover, fasting isoleucine levels correlated positively with insulin AUC.

**Table 1:** Associations between amino acids and derivatives, and fatty acids, with fasting and postprandial indices of insulin sensitivity.

Metabolites		BASELINE INDICES									POSTPRANDIAL INDICES					
		HOMA-IR			Fasting Glucose			Fasting Insulin			AUC Glucose			AUC Insulin		
**Amino acid and derivatives**		r adjusted	p-value	β	r adjusted	p-value	β	r adjusted	p-value	β	r adjusted	p-value	β	r adjusted	p-value	β
Creatine (μM)					0.464	<0.01	0.533									
Proline (μM)		0.645	<0.01	0.658	0.609	<0.01	0.490	0.630	<0.01	0.648						
Isoleucine (μM)														0.730	<0.01	0.460
Leucine (μM)		0.761	0.01	0.368				0.760	0.01	0.388						
**Class**	**Fatty acids**															
SFA	Myristic acid (14:0, %)	0.516	0.01	0.549	0.520	0.01	0.450	0.489	0.01	0.524	0.527	<0.01	0.458			
	Palmitic acid (16:0, %)	0.681	<0.01	0.662				0.675	<0.01	0.662	0.729	<0.01	0.523	0.700	<0.01	0.623
	Stearic acid (18:0, %)				-0.536	<0.01	-0.455									
MUFA	Myristoleic acid (14:1n-5, %)	0.433	0.01	0.617	0.671	<0.01	0.687				0.486	<0.01	-0.407			
	Oleic acid (18:1n-9, %)				0.644	<0.01	0.458									
PUFA	Linoleic acid (18:2n-6, %)				-0.629	<0.01	-0.433									

Significance was set at p ≤ 0.01 using Bonferroni correction for multiple testing. Linear regressions were adjusted for age, sex, and BMI.

18:2n-6 (whose %PP change differed significantly between the 3 groups) showed a significant inverse relationship with fasting glucose. For the other measured FAs, the associations between SFAs and markers of glucose homeostasis tended to separate into two groups. On the one hand, 14 and 16-carbon SFAs (i.e., myristic acid (14:0) and 16:0) were positively correlated with HOMA-IR, fasting insulin, and glucose AUC, while 14:0 was also positively associated with fasting glucose and 16:0 with insulin AUC. On the other hand, the longer chain SFA 18:0 was inversely associated with fasting glucose. The MUFA myristoleic acid (14:1n-5) was positively correlated with HOMA-IR, fasting glucose and glucose AUC; and 18:1n-9 was positively associated with fasting glucose.

## Discussion

### General Summary

We have previously reported that during catabolic conditions (i.e., fasting), the MHO group differed from LH and MUO individuals. Specifically, circulating AA, FA, and inflammatory marker profiles in MHO individuals were intermediate to LH and MUO individuals [13, 14]. Building on these previous observations, the present study investigated for the first time the response to a high calorie Western meal within these distinct groups of individuals. This is highly relevant as this corresponds to a real-world meal typical of the Western diet, as opposed to clinical tolerance tests. Importantly, study participants received standardized lifestyle advice 48 h prior to the caloric challenge in order to minimize inter-individual lifestyle differences, which was previously shown to adequately normalize the human metabolome to reduce variations between people [17]. We acknowledge that the caloric challenge imposed with the fast-food meal may be significantly greater for lean individuals compared to obese individuals when considering their habitual dietary habits; however, imposing the same meal challenge in all subjects was essential in order to emphasize the differences in the responses between the MHO and MUO groups in comparison to LH individuals. Nevertheless, future studies could account for habitual caloric intake and standardize the increase in calories in accordance to each participant’s daily caloric intake. Our results showed that the response to the high calorie meal differed between the three groups. We found that MHO individuals, similar to LH individuals, had a preserved glycemic and insulinemic postprandial response compared to their MUO counterparts despite having the same BMI and body fat %. Moreover, we observed distinct AA and FA profiles following the caloric challenge, and identified metabolites that significantly correlated with an individual’s glycemic and insulinemic response.

### AAs and their Derivatives

We examined AAs and AA-derivatives at fasting and postprandially to evaluate the %PP change within LH, MHO, and MUO groups. Analyzing the high calorie meal by CE-MS showed that it contained detectable levels for the majority of AAs. Correspondingly, most of the metabolites experienced similar increases or decreases following the meal between the three groups (S2 Table). This aligns with previous studies showing that AA homeostasis was perturbed in anabolic states (i.e., postprandially) following a caloric challenge [6, 18–21]. For example, concordant with our observations, BCAAs were increased following the ingestion of different breads in healthy postmenopausal women [6], and following a standardized high calorie Big Mac meal in discordant obese and lean twins [20]. Here, our goal was to determine if postprandial alterations in the AA profile differed between individuals varying in cardiometabolic risk.

When studying the %PP change, several AAs significantly differed between the 3 groups (Fig 3). This included serine and cystine, which showed a similar reduced %PP change in MHO and MUO individuals compared to LH. This is interesting, as total cyst(e)ine levels were shown to be a predictor of obesity and insulin resistance in both children and adults [22, 23]. In contrast, asparagine and glutamine levels had an intermediate %PP change in MHO relative to those of LH and MUO individuals. Asparagine and glutamine are precursors to aspartic acid and glutamic acid, respectively, via deamination, which are subsequently used to replenish intermediates for the tricarboxylic acid (TCA) cycle. This is notable as the TCA cycle was shown to reach its optimal capacity early in the postprandial phase [19]; therefore, the different groups of individuals may potentially differ with regards to metabolite flux entering and/or leaving the TCA cycle. Interestingly, we previously showed that the TCA cycle was impaired in subcutaneous adipose tissue from obese individuals, but to a lesser extent in MHO relative to MUO individuals [13]. We and others have also shown that the decreased expression of a number of genes associated with the TCA cycle and BCAA catabolism in adipose tissue from obese individuals was associated with higher serum BCAA levels and insulin resistance [13, 24, 25]. As such, it is tempting to speculate that the variable circulating levels of the aforementioned AAs, for which the %PP change between the three groups followed the general pattern of LH > MHO > MUO at T120, may stem from differences in TCA cycle functionality.

We also observed that the carnitine-to-acetylcarnitine ratio differed significantly between LH and MHO individuals, while found at intermediate levels in MUO individuals. Interestingly, we observed a trend for a higher %PP change in this ratio in LH individuals compared to the obese groups (absolute value, Fig 3 and S2 Table). This is intriguing given that carnitine and acylcarnitines, and their ratio, were suggested to indicate a switch from a catabolic (i.e., fasting) state to an anabolic state (i.e., postprandial) following a number of different caloric challenges (e.g., a standard liquid diet, an OGTT, an oral lipid tolerance test [18]), and also for predicting OGTT changes after high-intensity interval training [26]. During fasting, reduced carnitine levels in blood indicate an increased cellular uptake, while the release of acetyl- and acyl-CoA into blood due to increased FA β-oxidation is buffered by increased acylcarnitines. Therefore, the catabolic state is reflected by an increase in acylcarnitines and the anabolic status is reflected by a decrease in acylcarnitines [18]. Our results are consistent with this phenomenon, and revealed the highest %PP change in the ratio for the LH group (Fig 3), suggesting their heightened ability to metabolically respond to the caloric challenge.

As it has been shown that AAs, and particularly the most potent insulinogenic BCAAs (i.e., leucine and isoleucine), correlate with indices of insulin sensitivity and could predict T2D [6, 27, 28], we evaluated associations between fasting AAs and parameters of insulin sensitivity before and after the caloric challenge. Fasting levels for isoleucine correlated significantly with both fasting insulin levels and insulin AUC (Table 1); while leucine associated with HOMA-IR and fasting insulin. These results are in agreement with previous works suggesting that BCAAs could be used as predictors of insulin resistance and as biomarkers of T2D development [29–31].

### Fatty acids

In parallel to changes in AA homeostasis, we also observed modifications in the FA profile after consumption of the standardized meal. Most of the FAs increased or decreased in a similar way between fasting and T120 in the three groups of individuals (S3 Table). However, we found that four FAs (16:1n-7, 18:2n-6, 18:3n-6, and 20:4n-6) had significantly different %PP changes (Fig 4). Interestingly, 16:1n-7 showed the same trend in MHO and MUO individuals in comparison to LH, while the other FAs revealed %PP changes that were similar between LH and MHO groups.

Recent evidence has shown relationships between FAs, insulin sensitivity indices, and T2D risk [32]; therefore, we evaluated if FAs were also associated with parameters of insulin sensitivity in our study. Of the four FAs mentioned above, we observed a positive correlation between fasting 16:1n-7 levels and glucose AUC, and inverse correlations between 18:2n-6 and fasting glucose. We also extended our association analyses to include all measured FAs. This allowed the link between the profiled FAs and indices of insulin sensitivity at fasting, as well as their potential association with glycemic and insulinemic postprandial responses, to be investigated. We found that fasting levels of SFAs 14:0 and 16:0 correlated positively with all parameters of fasting and postprandial insulin sensitivity, while longer-chain 18:0 was inversely correlated with fasting glucose. Moreover, we also observed a trend for inverse associations between 18:0 (r = -0.492, p = 0.02) and 20:0 (r = -0.533, p = 0.03) with glucose AUC (data not shown). This is relevant given that, according to our observations, shorter chain SFAs (14:0 and 16:0) were previously linked with an unhealthy cardiometabolic profile, compared to longer chain SFAs (18:0, 22:0 and 24:0) [33–35]. This was further reinforced by the fact that the shorter chain SFAs associated with HOMA-IR, while 18:0 and 20:0 did not. Our results also revealed inverse correlations between 18:2n-6 PUFAs and fasting glucose that supports previous data indicating that this n-6 PUFA is inversely associated with T2D risk [36, 37].

### Study Limitations

The T120 time point is optimal for assessing glucose and insulin responses, and although differences in TG and free FAs were reported by van Dijk *et al*. in the 2 h postprandial period [2], we acknowledge that longer follow-up times and additional time points would generate further information regarding lipid responses following the standardized meal. This will help provide insights into inter-individual variations in nutrient absorption and gut microbial co-metabolism [38], as well as the association between metabolites and overall cardiometabolic risk (notably when expanding metabolomic coverage to include organic acids and lipids). While our sample size may be construed as small, the robust clinical characterization of our study participants, the use of a standardized caloric challenge, and the fact that each individual serves as their own control ensures a high degree of confidence in our results.

## Conclusions

In conclusion, the present study showed that the glycemic and insulinemic postprandial responses were significantly different between individuals varying in cardiometabolic risk. MHO had a greater ability to adapt to the caloric challenge compared to their MUO counterparts, thereby highlighting their preserved insulin sensitivity. The targeted metabolomic and FA profiling approaches revealed that several metabolites differed significantly after the challenge. Additionally, we identified metabolites at baseline that should be further studied for their potential to predict an individual’s postprandial response and cardiometabolic risk, independent of BMI. Indeed, the positive correlations seen between fasting levels of isoleucine and both fasting insulin levels and insulin AUC, as well as the positive associations seen between leucine and both HOMA-IR and fasting insulin, show the high potential of BCAA to identify “at risk” obese individuals. Further, the fasting levels of 14:0, 16:0, and 18:0 show promise as distinct markers of fasting and/or postprandial insulin sensitivity. This highlights the added value of postprandial measurements and underscores the importance to identify “at risk” obese individuals that could benefit from tailored diet interventions.

## Supporting Information

S1 Table Study population characteristics. Data represented as mean ± SEM. LH, lean healthy; MHO, metabolically healthy obese; MUO, metabolically unhealthy obese; BMI, body mass index; BP, blood pressure; Total-c, total-cholesterol; LDL-c, low-density lipoprotein; HDL-c, high-density lipoprotein; TG, triglycerides; HbA1c, glycosylated haemoglobin; HOMA-IR, homeostatic model assessment for insulin resistance; HOMA%B, homeostatic model assessment for β-cell function. A non-parametric ANOVA Kruskal-Wallis followed by a post-hoc Mann-Whitney test was used to determine the significance between groups (p<0.05). *Adapted from Perreault et al*. *PLoS One*. *2014; 9(2)*: *e88539*.
(DOCX)

S2 Table Mean circulating concentrations of amino acid and derivatives at fasting and T120 min time points. Data represented as mean concentration ± SEM. LH, lean healthy; MHO, metabolically healthy obese; MUO, metabolically unhealthy obese. A non-parametric ANOVA Kruskal-Wallis followed by a post-hoc Mann-Whitney test was used to determine significance (p < 0.05). Significant % postprandial (%PP) changes are indicated in bold.
(DOCX)

S3 Table Mean circulating concentrations of fatty acids at fasting and T120 min time points. Data presented as mean relative percentage ± SEM. LH, lean healthy; MHO, metabolically healthy obese; MUO, metabolically unhealthy obese. A non-parametric ANOVA Kruskal-Wallis followed by a post-hoc Mann-Whitney test was used to determine significance (p < 0.05). Significant % postprandial (%PP) changes are indicated in bold.
(DOCX)
