# Supplementary material for: Metabolomics Reveals Metabolically Healthy and Unhealthy Obese Individuals Differ in their Response to a Caloric Challenge
Source: PLoS One. 2015 Aug 14;10(8):e0134613. doi: 10.1371/journal.pone.0134613 (PMC4537251; doi:10.1371/journal.pone.0134613)
Supplement: S1 Table — Data represented as mean ± SEM. LH, lean healthy; MHO, metabolically healthy obese; MUO, metabolically unhealthy obese; BMI, body mass index; BP, blood pressure; Total-c, total-cholesterol; LDL-c, low-density lipoprotein; HDL-c, high-density lipoprotein; TG, triglycerides; HbA1c, glycosylated haemoglobin; HOMA-IR, homeostatic model assessment for insulin resistance; HOMA%B, homeostatic model assessment for β-cell function. A non-parametric ANOVA Kruskal-Wallis followed by a post-hoc Mann-Whitney test was used to determine the significance between groups (p<0.05). Adapted from Perreault et al. PLoS One. 2014; 9(2): e88539. (DOCX) [file pone.0134613.s001.docx]

**Table S1: Study population characteristics**.

| **Parameters** | **LH**  mean ± SEM | **MHO**  mean ± SEM | **MUO**  mean ± SEM | **ANOVA**  (p-value) | **Post-hoc Mann-Whitney-Wilcoxon**  **group comparison (p-values)** | | |
| --- | --- | --- | --- | --- | --- | --- | --- |
|  |  |  |  |  | LH vs. MHO | LH vs. MUO | MHO vs. MUO |
| *Anthropometric measurements* | | | | | | | |
| Number of subjects | 10  (3 men, 7 women) | 10  (3 men, 7 women) | 10  (3 men, 7 women) |  |  |  |  |
| Age (yrs) | 51 ± 3 | 50 ± 4 | 48 ± 2 | 0.8418 |  |  |  |
| Weight (kg) | 61.9 ± 2.8 | 86.2 ± 3.4 | 92.7 ± 6.2 | 0.0004 | 0.0001 | 0.0003 | 0.5288 |
| Height (cm) | 167 ± 3 | 168 ± 3 | 167 ± 3 | 0.9299 |  |  |  |
| BMI (kg/m^2^) | 22.1 ± 0.6 | 30.6 ± 1.1 | 33.0 ± 1.9 | ­<0.0001 | <0.0001 | <0.0001 | 0.4813 |
| Waist circumference (cm) | 77 ± 3 | 98 ± 3 | 104 ± 5 | 0.0002 | 0.0007 | 0.0003 | 0.4359 |
| Hip circumference (cm) | 96 ± 1 | 109 ± 2 | 113 ± 4 | 0.0002 | 0.0003 | 0.0006 | 0.5960 |
| Waist-to-hip ratio | 0.80 ± 0.02 | 0.90 ± 0.03 | 0.92 ± 0.02 | 0.0075 | 0.0172 | 0.0046 | 0.5787 |
| Fat mass (%) | 27.4 ±2.7 | 39.8 ± 2.4 | 39.3 ± 2.4 | 0.0086 | 0.0039 | 0.0115 | 0.9397 |
| Fat mass (kg) | 16.7 ± 1.6 | 34.1 ± 2.4 | 36.4 ± 3.4 | <0.0001 | <0.0001 | <0.0001 | 0.7959 |
| Lean mass (%) | 72.6 ± 2.7 | 60.2 ± 2.4 | 60.8 ± 2.4 | 0.0086 | 0.0039 | 0.0115 | 0.9397 |
| Lean mass (kg) | 45.1 ± 3.1 | 52.0 ± 3.2 | 56.2 ± 4.4 | 0.1071 |  |  |  |
| *Clinical measurements* | | | | | | | |
| Systolic BP (mmHg) | 118 ± 4 | 128 ± 5 | 128 ± 4 | 0.1063 |  |  |  |
| Diastolic BP (mmHg) | 75 ± 3 | 82 ± 2 | 82 ± 2 | 0.0664 |  |  |  |
| Total-c (mmol/L) | 4.43 ± 0.30 | 4.26 ± 0.32 | 5.34 ± 0.23 | 0.0169 | 0.7054 | 0.0355 | 0.0073 |
| LDL-c (mmol/L) | 2.52 ± 0.25 | 2.39 ± 0.30 | 3.27 ± 0.19 | 0.0401 | 0.6842 | 0.0433 | 0.0232 |
| HDL-c (mmol/L) | 1.57 ± 0.08 | 1.17 ± 0.12 | 1.04 ± 0.05 | 0.0018 | 0.0256 | 0.0004 | 0.3634 |
| Total-c/HDL ratio | 2.85 ± 0.16 | 3.81 ± 0.22 | 5.17 ± 0.18 | <0.0001 | 0.0073 | 0.0002 | 0.0010 |
| TG (mmol/L) | 0.77 ± 0.05 | 1.54 ± 0.33 | 2.26 ± 0.23 | 0.0004 | 0.0311 | 0.0002 | 0.0524 |
| Fasting glucose (mmol/L) | 4.5 ± 0.2 | 5.0 ±0.1 | 5.3 ± 0.2 | 0.0243 | 0.0335 | 0.0171 | 0.5178 |
| Fasting insulin (pmol/L) | 37 ± 17 | 64 ± 9 | 118 ± 22 | 0.0025 | 0.0129 | 0.0030 | 0.0887 |
| HbA1c (%) | 5.37 ± 0.07 | 5.72 ± 0.08 | 5.73 ± 0.08 | 0.0051 | 0.0055 | 0.0066 | 0.8775 |
| HOMA-IR | 0.65 ± 0.28 | 1.18 ± 0.16 | 2.19 ± 0.42 | 0.0024 | 0.0143 | 0.0015 | 0.0892 |
| HOMA%B | 93.7 ± 33.1 | 105.9 ± 10.1 | 140.3 ± 17.9 | 0.0130 | 0.0337 | 0.0076 | 0.2176 |
