# Supplementary material for: Metabolomics Reveals Metabolically Healthy and Unhealthy Obese Individuals Differ in their Response to a Caloric Challenge
Source: PLoS One. 2015 Aug 14;10(8):e0134613. doi: 10.1371/journal.pone.0134613 (PMC4537251; doi:10.1371/journal.pone.0134613)
Supplement: S2 Table — Data represented as mean concentration ± SEM. LH, lean healthy; MHO, metabolically healthy obese; MUO, metabolically unhealthy obese. A non-parametric ANOVA Kruskal-Wallis followed by a post-hoc Mann-Whitney test was used to determine significance (p < 0.05). Significant % postprandial (%PP) changes are indicated in bold. (DOCX) [file pone.0134613.s002.docx]

**Table S2: Mean circulating concentrations of amino acid and derivatives at fasting and T120 min time points.**

| **METABOLITES** | **FASTING**  **(mean ± SEM)** | | | **POSTPRANDIAL**  **(mean ± SEM)** | | | **% POSTPRANDIAL CHANGE (%PP)** | | |  |  |  |  |
| --- | --- | --- | --- | --- | --- | --- | --- | --- | --- | --- | --- | --- | --- |
| ***Plasma amino acid and derivatives*** | **LH** | **MHO** | **MUO** | **LH** | **MHO** | **MUO** | **LH** | **MHO** | **MUO** | **ANOVA Kruskal-Wallis**  **(p-value)** | **Post hoc Mann-Whitney group comparison**  **(p-values)** | | |
|  |  |  |  |  |  |  |  |  |  |  | **LH vs. MUO** | **LH vs. MHO** | **MHO vs. MUO** |
| Alanine (µM) | 339 ± 30 | 446 ± 24 | 413 ± 34 | 451 ± 39 | 557 ± 31 | 456 ± 28 | 34.7 ± 8.1 | 26.2 ± 7.3 | 14.2 ± 8.1 | 0.19 |  |  |  |
| Arginine (µM) | 82.7 ± 4.8 | 95.6 ± 6.0 | 88.5 ± 9.7 | 100 ± 8 | 108 ± 5 | 95.2 ± 8.2 | 20.3 ± 3.1 | 15.3 ± 5.4 | 11.4 ± 6.6 | 0.21 |  |  |  |
| **Asparagine (µM)** | **56.9 ± 3.2** | **56.9 ± 3.1** | **56.2 ± 4.9** | **75.0 ± 6.7** | **64.2 ± 4.7** | **56.9 ± 3.7** | 30.9 ± 6.8 | 13.5 ± 6.5 | 5.15 ± 7.09 | **0.03** | **0.01** | **0.06** | **0.29** |
| Aspartic acid (µM) | 9.97 ± 0.22 | 9.82 ± 0.16 | 10.8 ± 0.4 | 9.81 ± 0.19 | 10.0 ± 0.2 | 10.3 ± 0.3 | -1.40 ± 1.72 | 2.19 ± 1.64 | -2.91 ± 3.18 | 0.36 |  |  |  |
| Asparagine-to-aspartic acid ratio | 5.73 ± 0.35 | 5.81 ± 0.32 | 5.28 ± 0.48 | 7.69 ± 0.74 | 6.46 ± 0.50 | 5.57 ± 0.46 | 33.5 ± 8.0 | 11.9 ± 7.6 | 8.29 ± 6.21 | 0.07 |  |  |  |
| Betaine (µM) | 51 ± 4.0 | 44.4 ± 4.7 | 38.5 ± 3.4 | 57.1 ± 4.1 | 52.4 ± 4.0 | 45.8 ± 2.8 | 12.8 ± 3.5 | 22.3 ± 6.9 | 28.5 ± 15.5 | 0.54 |  |  |  |
| Carnitine (µM) | 42.1 ± 4.90 | 72.5 ± 8.7 | 48.6 ± 4.3 | 49.9 ± 4.8 | 71.4 ± 6.1 | 54.5 ± 3.7 | 21.6 ± 6.4 | 2.26 ± 4.89 | 14.4 ± 4.0 | 0.11 |  |  |  |
| Acetylcarnitine (µM) | 37.2 ± 2.3 | 40.6 ± 4.4 | 33.2 ± 3.7 | 18.0 ± 1.6 | 22.4 ± 1.6 | 17.9 ± 1.5 | -51.7 ± 2.3 | -41.8 ± 4.1 | -43.4 ± 3.5 | 0.12 |  |  |  |
| **Carnitine-to-acetylcarnitine ratio** | **1.14 ± 0.13** | **1.79 ± 0.09** | **1.60 ± 0.19** | **2.81 ± 0.21** | **3.18 ± 0.14** | **3.18 ± 0.26** | **155 ± 15** | **82.3 ± 13.1** | **113 ± 21** | **0.02** | **0.06** | **0.01** | **0.26** |
| Propylcarnitine (µM) | 0.65 ± 0.07 | 0.84 ± 0.08 | 0.72 ± 0.07 | 0.73 ± 0.08 | 1.00 ± 0.07 | 0.84 ± 0.06 | 15.4 ± 10.2 | 23.0 ± 6.2 | 21.7 ± 6.2 | 0.34 |  |  |  |
| Citrulline (µM) | 27.6 ± 2.0 | 30.7 ± 1.6 | 29.3 ± 2.0 | 22.0 ± 1.5 | 24.7 ± 1.5 | 22.4 ± 1.2 | -19.8 ± 2.4 | -18.5 ± 4.6 | -21.9 ± 4.1 | 0.85 |  |  |  |
| Creatinine (µM) | 64.0 ± 4.3 | 73.6 ± 5.9 | 64.0 ± 4.5 | 66.2 ± 5.8 | 70.0 ± 5.1 | 63.3 ± 3.9 | 2.58 ± 4.07 | -3.63 ± 3.87 | 0.32 ± 4.31 | 0.49 |  |  |  |
| Creatine (µM) | 32.7 ± 3.8 | 49.4 ± 5.5 | 42.5 ± 6.2 | 45.1 ± 5.0 | 60.2 ± 7.0 | 48.5 ± 4.6 | 38.6 ± 3.7 | 22.7 ± 4.3 | 22.4 ± 8.7 | 0.06 |  |  |  |
| **Cystine (µM)** | **46.0 ± 2.3** | **61.5 ± 5.5** | **52.5 ± 3.0** | **49.9 ± 2.1** | **58.6 ± 3.1** | **49.5 ± 3.2** | **9.23 ± 3.24** | **-2.31 ± 4.02** | **-5.72 ± 2.82** | **0.02** | **<0.01** | **0.04** | **0.52** |
| Cysteine-glutathione disulfide (µM) | 9.32 ± 1.13 | 8.06 ± 0.62 | 7.97 ± 0.77 | 7.76 ± 0.40 | 7.52 ± 0.35 | 6.86 ± 0.21 | -11.2 ± 7.7 | -3.96 ± 5.07 | -9.00 ± 5.79 | 0.46 |  |  |  |
| Deoxycarnitine (µM) | 1.37 ± 0.14 | 1.83 ± 0.16 | 1.27 ± 0.13 | 1.28 ± 0.14 | 1.73 ± 0.17 | 1.23 ± 0.13 | -6.86 ± 3.84 | -5.64 ± 4.16 | -2.66 ± 4.51 | 0.74 |  |  |  |
| Dimethylglycine (µM) | 8.44 ± 0.25 | 8.17 ± 0.16 | 9.01 ± 0.29 | 8.67 ± 0.27 | 8.33 ± 0.21 | 8.89 ± 0.21 | 2.83 ± 2.17 | 1.92 ± 1.34 | -0.77 ± 2.50 | 0.68 |  |  |  |
| γ-Amino-butyric acid (µM) | 6.70 ± 0.13 | 6.34 ± 0.09 | 6.76 ± 0.23 | 6.35 ± 0.14 | 6.16 ± 0.07 | 6.40 ± 0.20 | -5.12 ± 1.39 | -2.74 ± 1.22 | -5.15 ± 1.29 | 0.38 |  |  |  |
| **Glutamine (µM)** | **537 ± 16** | **545 ± 20** | **510 ± 30** | **587 ± 27** | **570 ± 31** | **473 ± 20** | **9.33 ± 4.08** | **4.61 ± 4.44** | **-6.06 ± 3.40** | **0.03** | **0.01** | **0.46** | **0.07** |
| Glutamic acid (µM) | 33.7 ± 4.1 | 40.4 ± 3.9 | 51.5 ± 5.0 | 34.5 ± 3.7 | 36.2 ± 2.4 | 49.3 ± 7.5 | 5.23 ± 9.11 | -3.39 ± 9.58 | -3.68 ± 9.27 | 0.83 |  |  |  |
| Gutamine-to-glutamic acid ratio | 17.5 ± 1.8 | 14.7 ± 1.6 | 11.2 ± 1.6 | 18.9 ± 2.4 | 16.5 ± 1.7 | 11.4 ± 1.4 | 11.1 ± 10.9 | 20.6 ± 14.6 | 8.92 ± 14.7 | 0.77 |  |  |  |
| Glycine (µM) | 209 ± 18 | 204 ± 18 | 165 ± 14 | 226 ± 21 | 207 ± 21 | 153 ± 13 | 7.32 ± 4.21 | 1.09 ± 3.95 | -5.84 ± 4.32 | 0.12 |  |  |  |
| Histidine (µM) | 85.1 ± 3.4 | 95.8 ± 5.8 | 90.9 ± 5.3 | 95.3 ± 3.9 | 98.2 ± 6.4 | 90.5 ± 3.8 | 12.2 ± 2.1 | 3.42 ± 5.25 | 1.05 ± 3.70 | 0.06 |  |  |  |
| Hydroxyproline (µM) | 15.1 ± 0.5 | 18.0 ± 0.6 | 15.9 ± 0.9 | 17.9 ± 0.6 | 19.5 ± 0.47 | 17.5 ± 0.8 | 18.9 ± 3.3 | 8.76 ± 3.17 | 11.59 ± 4.07 | 0.13 |  |  |  |
| Hydroxyxanthine (µM) | 4.14 ± 0.93 | 3.97 ± 0.29 | 3.84 ± 0.43 | 2.79 ± 0.35 | 2.54 ± 0.20 | 2.79 ± 0.19 | -22.4 ± 10.6 | -34.7 ± 4.2 | -22.4 ± 6.6 | 0.18 |  |  |  |
| Isoleucine (µM) | 49.2 ± 2.6 | 59.2 ± 3.3 | 62.2 ± 4.4 | 64.6 ± 3.4 | 70.4 ± 3.5 | 74.7 ± 4.7 | 31.9 ± 3.8 | 21.2 ± 6.5 | 23.3 ± 8.0 | 0.34 |  |  |  |
| Kynurenic acid (µM) | 3.03 ± 0.08 | 2.99 ± 0.08 | 3.17 ± 0.05 | 2.98 ± 0.10 | 2.95 ± 0.10 | 3.13 ± 0.05 | -1.51 ± 1.99 | -1.58 ± 2.15 | -0.88 ± 1.49 | 0.85 |  |  |  |
| Leucine (µM) | 96.5 ± 6.0 | 117 ± 7.0 | 121 ± 12 | 114 ± 7 | 125 ± 6 | 129 ± 9 | 19.6 ± 2.4 | 8.40 ± 5.69 | 12.1 ± 9.7 | 0.25 |  |  |  |
| Lysine (µM) | 129 ± 7 | 179 ± 11 | 151 ± 13 | 144 ± 7 | 179 ± 8 | 158 ± 13 | 11.6 ± 3.4 | 1.59 ± 3.28 | 4.82 ± 3.77 | 0.15 |  |  |  |
| Methionine (µM) | 24.4 ± 1.0 | 27.8 ± 1.6 | 27.1 ± 1.9 | 30.5 ± 2.2 | 30.5 ± 1.3 | 28.4 ± 1.6 | 25.0 ± 7.7 | 11.5 ± 4.5 | 6.84 ± 5.88 | 0.23 |  |  |  |
| 3-Methylhistidine (µM) | 21.9 ± 1.3 | 29.7 ± 2.7 | 22.3 ± 1.7 | 20.5 ± 1.1 | 26.1 ± 2.1 | 20.9 ± 1.7 | -6.04 ± 1.7 | -10.3 ± 3.9 | -6.43 ± 1.46 | 0.90 |  |  |  |
| Ornithine (µM) | 43.3 ± 2.1 | 56.0 ± 4.9 | 50.2 ± 4.8 | 52.2 ± 2.6 | 63.5 ± 4.4 | 56.9 ± 4.8 | 21.4 ± 5.9 | 15.8 ± 5.7 | 15.3 ± 4.4 | 0.73 |  |  |  |
| Phenylalanine (µM) | 52.6 ± 1.7 | 60.9 ± 3.3 | 58.8 ± 3.4 | 66.6 ± 2.7 | 70.3 ± 2.1 | 67.5 ± 2.1 | 26.9 ± 4.5 | 17.1 ± 4.6 | 16.7 ± 5.1 | 0.30 |  |  |  |
| Proline (µM) | 130 ± 11 | 163 ± 10 | 153 ± 15 | 215 ± 12 | 254 ± 11 | 226 ± 15 | 70.5 ± 10.6 | 59.2 ± 8.7 | 52.8 ± 8.6 | 0.54 |  |  |  |
| **Serine (µM)** | **120 ± 6** | **123 ± 12** | **103 ± 7** | **139 ± 10** | **124 ± 11** | **96.7 ± 5.8** | **14.6 ± 4.2** | **2.99 ± 5.53** | **-4.34 ± 5.04** | **0.03** | **0.03** | **0.02** | **0.36** |
| Threonine (µM) | 133 ± 11 | 147 ± 11 | 123 ± 11 | 147 ± 15 | 146 ± 11 | 117 ± 8 | 9.53 ± 3.40 | 0.12 ± 4.41 | -2.90 ± 4.42 | 0.10 |  |  |  |
| Tryptophan (µM) | 53.7 ± 2.6 | 58.7 ± 2.9 | 57.1 ± 3.4 | 59.0 ± 3.2 | 61.9 ± 2.8 | 57.4 ± 2.6 | 9.91 ± 3.00 | 6.53 ± 4.40 | 1.56 ± 2.85 | 0.27 |  |  |  |
| Tyrosine (µM) | 56.4 ± 3.3 | 71.3 ± 3.8 | 73.7 ± 7.3 | 62.9 ± 3.6 | 77.3 ± 2.5 | 74.9 ± 5.3 | 12.3 ± 4.05 | 9.85 ± 4.15 | 4.28 ± 6.03 | 0.80 |  |  |  |
| Valine (µM) | 210 ± 16 | 267 ± 15 | 256 ± 22 | 238 ± 17 | 284 ± 18 | 296 ± 28 | 13.8 ± 3.06 | 7.04 ± 4.34 | 17.4 ± 8.9 | 0.77 |  |  |  |
