# Supplementary material for: Metabolomics Reveals Metabolically Healthy and Unhealthy Obese Individuals Differ in their Response to a Caloric Challenge
Source: PLoS One. 2015 Aug 14;10(8):e0134613. doi: 10.1371/journal.pone.0134613 (PMC4537251; doi:10.1371/journal.pone.0134613)
Supplement: S3 Table — Data presented as mean relative percentage ± SEM. LH, lean healthy; MHO, metabolically healthy obese; MUO, metabolically unhealthy obese. A non-parametric ANOVA Kruskal-Wallis followed by a post-hoc Mann-Whitney test was used to determine significance (p < 0.05). Significant % postprandial (%PP) changes are indicated in bold. (DOCX) [file pone.0134613.s003.docx]

**Table S3: Mean circulating concentrations of fatty acids at fasting and T120 min time points.**

| **METABOLITES** | **FASTING**  **(mean ± SEM)** | | | **POSTPRANDIAL**  **(mean ± SEM)** | | | **% POSTPRANDIAL CHANGE (%PP)** | | |  |  | |  |  | |
| --- | --- | --- | --- | --- | --- | --- | --- | --- | --- | --- | --- | --- | --- | --- | --- |
| ***Serum fatty acids (%)*** | **LH** | **MHO** | **MUO** | **LH** | **MHO** | **MUO** | **LH** | **MHO** | **MUO** | **ANOVA Kruskal-Wallis**  **(p-value)** | **Post hoc Mann-Whitney group comparison**  **(p-values)** | | | | |
|  |  |  |  |  |  |  |  |  |  |  | **LH vs. MUO** | **LH vs. MHO** | | | **MHO vs. MUO** |
| Myristic acid  (14:0) | 0.62 ± 0.04 | 0.84 ± 0.08 | 1.20 ± 0.11 | 0.84 ± 0.04 | 0.93 ± 0.09 | 1.30 ± 0.10 | 38.0 ± 12.0 | 12.0 ± 6.0 | 10.0 ± 4.00 | 0.15 |  |  | | |  |
| Pentadecanoic acid  (15:0) | 0.23 ± 0.01 | 0.2 0 ± 0.01 | 0.23 ± 0.01 | 0.25 ± 0.01 | 0.24 ± 0.02 | 0.23 ± 0.01 | 8.00 ± 5.00 | 15.0 ± 11.0 | -1.00 ± 4.00 | 0.52 |  |  | | |  |
| Palmitic acid  (16:0) | 20.2 ± 0.4 | 21.2 0.6 | 22.2 ± 0.64 | 21.7 ± 0.4 | 22.0 ± 0.5 | 23.7 ± 0.7 | 7.00 ± 1.00 | 3.00 ± 1.00 | 6.00 ± 1.00 | 0.11 |  |  | | |  |
| Stearic acid  (18:0) | 7.71 ± 0.18 | 7.50 ± 0.27 | 6.73 ± 0.18 | 8.02 ± 0.11 | 7.64 ± 0.2 | 6.88 ± 0.16 | 5.00 ± 1.00 | 2.00 ± 2.00 | 2.00 ± 2.00 | 0.55 |  |  | | |  |
| Nonadecanoic acid  (19:0) | 0.05 ± 0.01 | 0.07 ± 0.01 | 0.05 ± 0.01 | 0.17 ± 0.04 | 0.13 ± 0.02 | 0.08 ± 0.01 | 164 ± 60 | 170 ± 58 | 71.0 ± 31.0 | 0.36 |  |  | | |  |
| Arachidic acid  (20:0) | 0.29 ± 0.03 | 0.20 ± 0.02 | 0.19 ± 0.01 | 0.26 ± 0.04 | 0.16 ± 0.02 | 0.17 ± 0.01 | -3.00 ± 21.0 | -21.0 ± 6.0 | -9.00 ± 7.00 | 0.48 |  |  | | |  |
| Behenic acid  (22:0) | 0.46 ± 0.03 | 0.39 ± 0.05 | 0.35 ± 0.04 | 0.38 ± 0.02 | 0.31 ± 0.07 | 0.29 ± 0.04 | -10.0 ± 7.00 | -12.0 ± 15.0 | -9.00 ± 10.0 | 0.96 |  |  | | |  |
| Lignoceric acid  (24:0) | 2.65 ± 0.31 | 2.37 ± 0.31 | 1.80 ± 0.10 | 1.75 ± 0.28 | 1.85 ± 0.27 | 1.58 ± 0.10 | -24.0 ± 10.0 | -12.0 ± 11.0 | -12.0 ± 2.00 | 0.49 |  |  | | |  |
| Myristoleic acid  (14:1n-5) | 0.06 ± 0.01 | 0.08 ± 0.01 | 0.11 ± 0.01 | 0.05 ± 0.01 | 0.08 ± 0.01 | 0.08 ± 0.01 | -1.00 ± 8.00 | -1.00 ± 7.0 | -13.0 ± 10.0 | 0.38 |  |  | | |  |
| **Palmitoleic acid**  **(16:1n-7)** | **1.57 ± 0.13** | **2.16 ± 0.25** | **2.33 ± 0.14** | **1.42 ± 0.11** | **2.02 ± 0.22** | **2.23 ± 0.1** | **-11.0 ± 2.00** | **-5.00 ± 3.00** | **-3.00 ± 1.00** | **0.04** | **0.04** | **0.02** | | | **0.73** |
| Heptadecanoic acid  (17:1n-7) | 0.14 ± 0.01 | 0.19 ± 0.02 | 0.20 ± 0.01 | 0.12 ± 0.02 | 0.11 ± 0.02 | 0.15 ± 0.03 | -11.0 ± 14.0 | -18.0. ± 15.0 | -40.0 ± 12.0 | 0.22 |  |  | | |  |
| Vaccenic acid  (18:1n-7) | 1.81 ± 0.07 | 1.88 ± 0.06 | 1.77 ± 0.04 | 1.89 ± 0.06 | 1.93 ± 0.07 | 1.83 ± 0.05 | 1.00 ± 1.00 | 2.00 ± 2.00 | 3.00 ± 1.00 | 0.46 |  |  | | |  |
| Oleic acid  (18:1n-9) | 19.6 ± 0.5 | 21.7 ± 1.2 | 23.7 ± 0.4 | 21.3 ± 0.7 | 22.5 ± 1.29 | 25.4 ± 0.5 | 8.00 ± 2.00 | 3.00 ± 2.00 | 7.00 ± 1.00 | 0.79 |  |  | | |  |
| cis-Nonadecanoic acid  (19:1n-9) | 0.28 ± 0.05 | 0.27 ± 0.04 | 0.15 ± 0.01 | 0.27 ± 0.07 | 0.25 ± 0.06 | 0.16 ± 0.03 | 19.0 ± 25.0 | 28.0 ± 43.0 | 10.0 ± 25.0 | 0.84 |  |  | | |  |
| Erucic acid  (22:1n-9) | 0.56 ± 0.06 | 0.58 ± 0.07 | 0.36 ± 0.03 | 0.53 ± 0.17 | 0.46 ± 0.14 | 0.25 ± 0.08 | -13.0 ± 19.0 | -8.00 ± 28.0 | -32.0 ± 18.0 | 0.84 |  |  | | |  |
| Nervonic acid  (24:1n-9) | 0.07 ± 0.01 | 0.06 ± 0.01 | 0.04 ± 0.01 | 0.05 ± 0.01 | 0.04 ± 0.01 | 0.04 ± 0.01 | -22.0 ± 11.0 | -24.0 ± 8.0 | 11.0 ± 32.0 | 0.94 |  |  | | |  |
| **Linoleic acid**  **(18:2n-6)** | **30.4 ± 0.8** | **25.7 ± 0.9** | **26.5 ± 1.0** | **28.7 ± 0.4** | **25.1 ± 1.0** | **24.8 ± 1.0** | **-4.4 ± 2.00** | **-2.20 ± 1.00** | **-6.00 ± 1.00** | **0.04** | **0.66** | **0.13** | | | **0.01** |
| **γ-linolenic acid**  **(18:3n-6)** | **0.36 ± 0.04** | **0.60 ± 0.06** | **0.55 ± 0.04** | **0.40 ± 0.03** | **0.61 ± 0.07** | **0.49 ± 0.04** | **9.00 ± 11.0** | **1.00 ± 5.00** | **-12.0 ± 2.00** | **0.04** | **0.02** | **0.90** | | | **0.04** |
| α-linolenic acid  (18:3n-3) | 0.70 ± 0.05 | 0.71 ± 0.05 | 0.95 ± 0.05 | 0.82 ± 0.04 | 0.74 ± 0.06 | 1.01 ± 0.05 | 19.0 ± 9.00 | 4.80 ± 4.00 | 7.00 ± 1.00 | 0.78 |  |  | | |  |
| Eicosadienoic acid  (20:2n-6) | 0.16 ± 0.02 | 0.13 ± 0.01 | 0.12 ± 0.01 | 0.16 ± 0.02 | 0.19 ± 0.03 | 0.20 ± 0.02 | 31.0 ± 15.0 | 74.0 ± 43.0 | 68.0 ± 29.0 | 0.74 |  |  | | |  |
| Dihomo-γ-linolenic acid (20:3n-6) | 1.47 ± 0.14 | 1.79 ± 0.09 | 1.64 ± 0.11 | 1.30 ± 0.10 | 1.57 ± 0.10 | 1.37 ± 0.10 | -16.0 ± 2.00 | -12.0 ± 2.00 | -16.0 ± 2.00 | 0.21 |  |  | | |  |
| **Arachidonic acid**  **(20:4n-6)** | **7.38 ± 0.52** | **8.31 ± 0.66** | **6.07 ± 0.36** | **6.72 ± 0.40** | **8.12 ± 0.66** | **5.31 ± 0.30** | **-11.0 ± 2.00** | **-1.00 ± 5.00** | **-12.0 ± 2.00** | **0.04** | **0.6** | **0.06** | | | **0.02** |
| Eicosapentaenoic acid  (20:5n-3) | 0.95 ± 0.16 | 0.91 ± 0.09 | 0.85 ± 0.11 | 0.73 ± 0.11 | 0.90 ± 0.10 | 0.78 ± 0.09 | -9.00 ± 5.00 | -1.00 ± 3.00 | -6.00 ± 3.00 | 0.21 |  |  | | |  |
| Adrenic acid  (22:4n-6) | 0.22 ± 0.02 | 0.20 ± 0.02 | 0.15 ± 0.02 | 0.18 ± 0.01 | 0.17 ± 0.01 | 0.13 ± 0.01 | -11.0 ± 10.0 | -10.0 ± 9.00 | -4.00 ± 9.00 | 0.83 |  |  | | |  |
| Docosapentaenoic acid  (22:5n-3) | 0.69 ± 0.03 | 0.64 ± 0.05 | 0.59 ± 0.02 | 0.61 ± 0.03 | 0.60 ± 0.04 | 0.56 ± 0.03 | -12.0 ± 2.00 | -4.00 ± 2.00 | -5.00 ± 4.00 | 0.11 |  |  | | |  |
| Docosapentaenoic acid  (22:5n-6) | 0.21 ± 0.01 | 0.20 ± 0.01 | 0.18 ± 0.01 | 0.17 ± 0.01 | 0.19 ± 0.01 | 0.17 ± 0.01 | -13.0 ± 5.00 | -3.00 ± 6.00 | -5.00 ± 6.00 | 0.49 |  |  | | |  |
| Docosahexaenoic acid  (22:6n-3) | 0.77 ± 0.04 | 0.66 ± 0.07 | 0.52 ± 0.06 | 0.77 ± 0.05 | 0.71 ± 0.07 | 0.50 ± 0.06 | 1.00 ± 7.00 | 11.0 ± 10.0 | -4.00 ± 6.00 | 0.46 |  |  | | |  |
